# Extracorporeal photopheresis therapy rapidly changes the cytokine profile and tumor microenvironment in cutaneous T cell lymphoma

**DOI:** 10.3389/fimmu.2025.1669015

**Published:** 2025-09-25

**Authors:** Susanne Melchers, Luisa Tengler, Özge Ç. Şener, Paul L. Beltzig, Ronny Schmidt, Stefan Klein, Jochen S. Utikal, Jan P. Nicolay

**Affiliations:** ^1^ Department of Dermatology, Venereology and Allergology, University Medical Center Mannheim/University of Heidelberg, Mannheim, Germany; ^2^ Skin Cancer Unit, German Cancer Research Center (DKFZ), Heidelberg, Germany; ^3^ Section of Clinical and Experimental Dermatology, Medical Faculty Mannheim, University of Heidelberg, Mannheim, Germany; ^4^ Sciomics GmbH, Heidelberg, Germany; ^5^ Department of Hematology and Oncology, Medical Faculty Mannheim, University of Heidelberg, Mannheim, Germany; ^6^ DKFZ-Hector Cancer Institute at the University Medical Center Mannheim, Mannheim, Germany

**Keywords:** cutaneous T cell lymphoma, extracorporeal photopheresis, Sézary syndrome, graft-versus-host-disease, transimmunization

## Abstract

Primary cutaneous T cell lymphomas (CTCL) are a heterogeneous group of rare lymphoproliferative disorders originating in the skin. Extracorporeal photopheresis (ECP) is an established, effective and excellently tolerable CTCL therapy, that can also be applied for the treatment of graft vs. host disease (GvHD). However, the underlying molecular mechanisms of ECP have not yet been fully clarified and seem to be dependent on the underlying disease. In this study, peripheral blood samples collected from six CTCL and three GvHD patients were analyzed pre- and post-ECP within one treatment of ECP for short-term alterations in the cytokine and chemokine milieu in the plasma and the composition of the peripheral blood mononuclear cell (PBMC) subsets. In CTCL, the plasma profiling revealed a lower expression of IL-15, IL-17, ICOS and higher expression of IL-13 post-ECP compared to the pre-ECP samples. Additionally, ECP led to an increased expression of the cell death inducers Fas and TRAIL. Flow cytometry revealed a significant increase in the CD14+ monocytes post-ECP in the CTCL patients, and a tendency of higher CD3+CD4- cytotoxic T cells in GvHD patient. Therefore, one cycle of ECP can induce detectable alterations in the peripheral blood of both CTCL and GvHD patients. This study contributes to the elucidation of the molecular mechanisms of ECP therapy and the detection of potential biomarkers for therapeutic response to ECP.

## 1 Introduction

Primary cutaneous T cell lymphomas (CTCL) represent a heterogeneous group of lymphoproliferative disorders originating in the skin that may subsequently progress to other compartments like the lymph nodes, viscera and the peripheral blood. Sézary Syndrome (SS) is a leukemic CTCL variant that is characterized by the detection of a clonal, malignant cell population in the peripheral blood, lymph node involvement, and erythroderma (1–3). SS is considered the highest CTCL stage according to the WHO-EORTC classification, and the prognosis is affected with a reported 5-year survival rate of 36% (4, 5).

To date, treatment of SS poses a therapeutic challenge due to high relapse rates and therapy resistance to initially highly effective therapies (6). Currently, only allogenic stem cell transplantation (alloSCT) poses a potentially curative therapeutic approach. However, due to high mortality and morbidity, alloSCT is currently only available to a limited patient collective (7). Therefore, sequential and combinatory therapy regimes are increasingly administered to increase the “time to next treatment” (TTNT), the time to the start of the next systemic CTCL therapy (8, 9). The overall response rate to ECP in CTCL patients varies from 54% to 74% with a complete response rate of 14% to 33.3% (10). It emerged that SS patients particularly benefit from an early inclusion of the extracorporeal photopheresis (ECP) in their combinatory therapeutic regimens since ECP increased the TTNT (11).

During ECP, the peripheral blood is UV-sensitized by the administration of 8-Methoxy psoralene (8-MOP) and treated extracorporeally with UVA irradiation. ECP was established by Edelson et al. in 1976 and is since in clinical use for the treatment of CTCL (12). Additionally, ECP is licensed for the treatment of GvHD, systemic sclerosis, and solid organ transplant rejection, and showed beneficial effects in immune-related colitis under immune-checkpoint-blockade (13–17). Although its efficacy and excellent safety profile is undisputed, the exact mode of action of ECP has not yet been comprehensively. Early reports from 1992 postulate that ECP induces the production of TNF-α by monocytes and that this effect is mediated by IFN-γ (18). In 1997 it was discovered that ECP restores the Th1/Th2 imbalance in CTCL patients by measuring IL-4, IL-12, and IFN-γ alterations upon ex vivo ECP treatment (19). This effect can also be observed in GvHD patients. Strikingly, ECP restores the Th1/Th2 imbalance in CTCL towards a more pro-inflammatory Th1 milieu, while in GvHD, ECP manages to promote a Th2 milieu.

In this study, we investigate the effects of ECP on both the cytokine and chemokine milieu, and the peripheral blood mononuclear cell populations pre- and post-ECP both in samples collected from human CTCL and GvHD patients in order to contribute to the elucidation of the underlying molecular mechanisms of ECP.

## 2 Materials and methods

### 2.1 Patients

Six SS patients diagnosed according to WHO-EORTC classification of CTCL and criteria of the International Society of Cutaneous Lymphomas (ISCLC) were included in the study (1, 3, 20). Additionally, three GvHD patients and three healthy donors were analyzed. Peripheral blood samples were collected and PBMCs, and plasma, were isolated as described before (21, 22). The clinical data of the patients are provided in **Supplementary Table S1**. Written informed consent was obtained from all patients. The study was conducted according to ethical guidelines at our institution and the Helsinki Declaration and was approved by the ethics committee II of the University of Heidelberg (reference number 2018-653N-MA).

### 2.2 Cytokine profiling

In total, 12 plasma samples collected from six SS patients before and after the application of one ECP treatment were analyzed. The bulk protein concentration was determined by BCA assay. A reference sample was established by pooling an identical volume of each sample using all study samples. The samples were labelled at an adjusted protein concentration for two hours with scioDye 2 (Sciomics, Neckargemünd, Germany). The reference sample was labelled with scioDye 1 (Sciomics, Neckargemünd, Germany). After two hours the reaction was stopped and the buffer exchanged to PBS. All labelled protein samples were stored at -20 °C until use. In total, 12 samples were analysed in a dual-color approach using a reference-based design on 12 scioCD antibody microarrays (Sciomics, Neckargemünd, Germany) targeting different CD surface markers and cytokines/chemokines. Each antibody is represented on the array in four replicates. The arrays were blocked with scioBlock (Sciomics, Neckargemünd, Germany) on a Hybstation 4800 (Tecan, Crailsheim, Germany) and afterwards the samples were incubated competitively with the reference sample using a dual-colour approach. After incubation for three hours, the slides were thoroughly washed with 1x PBST-T, rinsed with 0.1x PBS as well as with water and subsequently dried with nitrogen.

### 2.3 Data acquisition and analysis of the cytokine profiling by sciomics

Slide scanning was conducted using a Powerscanner (Tecan, Crailsheim, Germany) with constant instrument laser power and PMT settings. Spot segmentation was performed with GenePix Pro 6.0 (Molecular Devices, Union City, U.S.). Acquired raw data were analyzed using the linear models for microarray data (LIMMA) package of R-Bioconductor after uploading the median signal intensities. For normalization, a specialized invariant Lowess method was applied.

For analysis of the samples, a multi-factorial linear model was fitted via least squares regression with LIMMA, resulting in a two-sided t-test or F-test based on moderated statistics. Next to main factor (timepoint), the patient origin of each sample was accounted for using the information as an additional factor in the linear model. All presented p values were adjusted for multiple testing by controlling the false discovery rate according to Benjamini and Hochberg. Differences in protein abundance between different samples or sample groups are presented as log-fold changes (logFC) calculated for the basis 2. In a study comparing samples versus control a logFC = 1 means that the sample group had on average a 2^1^ = 2-fold higher signal than the control group. logFC = −1 stands for 2^−1^ = 1/2 of the signal in the sample as compared to the control group. Proteins with a |logFC| > 0.5 and an adjusted p value < 0.05 were defined as differential and displayed in blue in the following volcano plots. Proteins reaching reduced thresholds as defined individually for each comparison, are defined as noteworthy and displayed in green.

### 2.4 Flow cytometry

Frozen PBMC samples were thawed quickly in RPMI + 10% FCS. Cells were treated with Brefeldin A (Thermo Fisher Scientific, Dreieich, Germany) and incubated for 6 hours to block protein transport for subsequent intracellular cytokine detection (Panel 1). Following incubation (Panel 1) or directly after thawing (Panel 2), cells were blocked using Fc receptor blocking reagent (Miltenyi Biotech, Bergisch Gladbach, Germany) and stained in Brilliant Stain Buffer (BD, Heidelberg, Germany) using distinct multicolor flow cytometry panels. Both panels shared a common set of surface markers: CD19-AF488, CD4-AF488, CD3-AF700, CD56-BV785, CD14-RB670, CD26-BV421, and CD45-V500. Panel 2 additionally included ICOS-PE-Cy7, Fas-PE, CD163-APC and FasL-BV650 (**Supplementary Table S2**). Cells were stained for 30 minutes at 4°C. After the staining, cells were washed twice. Samples stained with Panel 2 were measured immediately acquiring 100,000 cells per sample with the NovoCyte 3005 flow cytometer (OLS, Bremen, Germany). Cells stained with Panel 1 underwent fixation with Cytofix/Cytoperm buffer (BD, Heidelberg, Germany) for 20 minutes at 4°C, followed by permeabilization using Perm/Wash buffer (BD, Heidelberg, Germany). Intracellular staining with IL-4-PE, IL-13-PE-Cy7 and TNF-α-APC was then performed for 30 minutes at 4°C. After two additional washes, 100,000 cells were acquired per sample. Compensation was done using stained cells and compensation beads (BioLegend, Amsterdam, Netherlands). Fluorescence minus one (FMO) controls were performed to define gating and avoid spectral overlap. The gating strategy is provided in the supplements (**Supplementary Figure S1**).

### 2.5 Statistics

The statistical analyses were calculated with GraphPad Prism (GraphPad Software, San Diego, U.S.). The differences were considered significant at p < 0.05, and the level of significance is indicated by asterisks (* p ≤ 0.05).

## 3 Results

### 3.1 One ECP treatment induces differential protein expression in the plasma in CTCL patients

**Figure 1:**
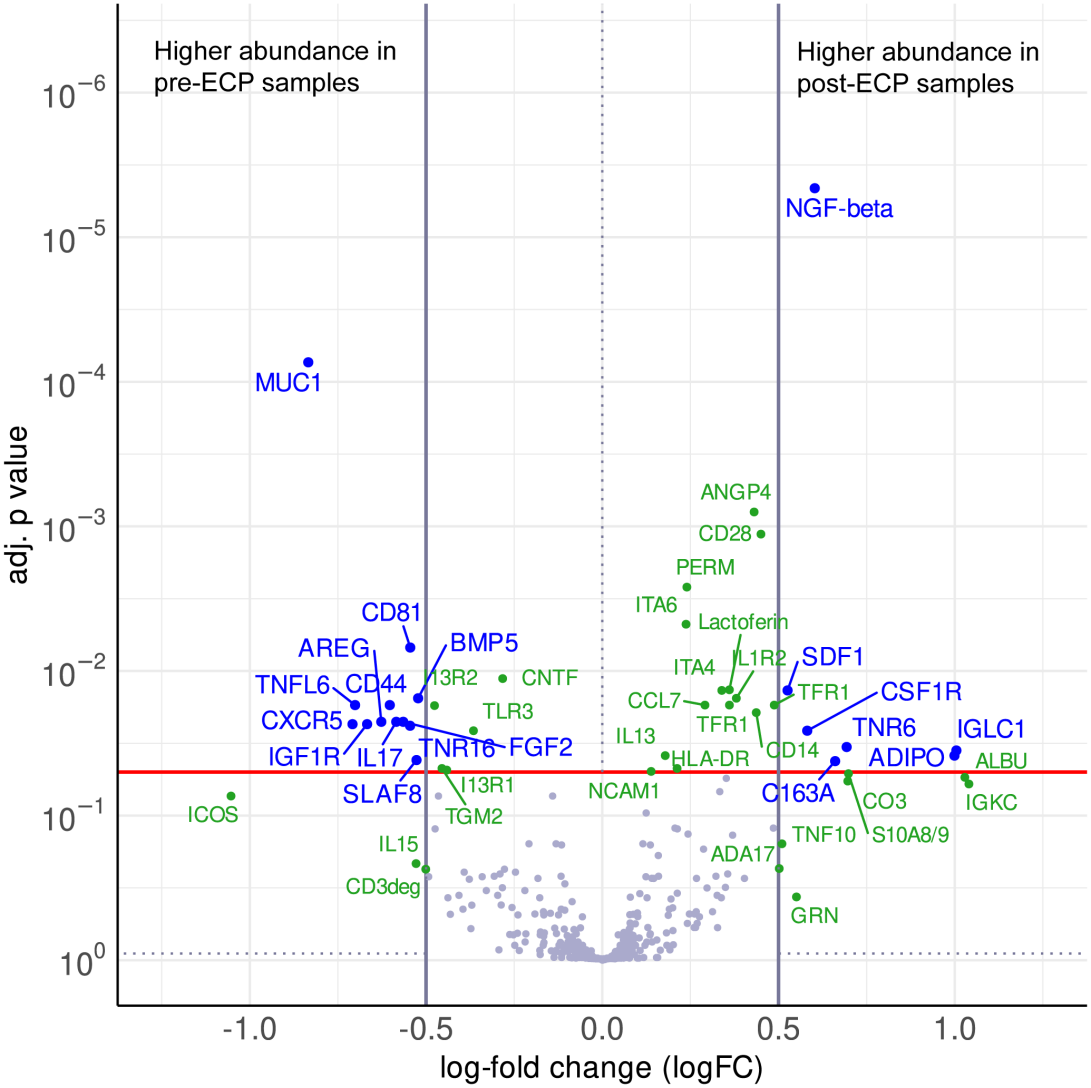
Volcano plot visualizing the differences in abundance between post-ECP samples and pre-ECP samples as log-fold changes (logFC) and their corresponding p values (adj. for multiple testing). The red line indicates a significance level of adj. p value = 0.05, vertical lines indicate the logFC cutoffs of ±0.5. A positive logFC indicates higher abundance in post-ECP samples, a negative logFC in pre-ECP samples. Differential proteins (|logFC| > 0.5, adj. p value < 0.05) are displayed with blue names. Non-significant proteins (adj. p value < 0.9) with a |logFC| > 0.5, as well as significant proteins which do not reach the logFC threshold are defined as noteworthy and displayed with green names.

Between the pre-ECP samples and the post-ECP samples, the cytokine profiling revealed a differential or noteworthy protein abundance in the plasma of CTCL patients. 19 antibodies detected a differential protein abundance, while 29 antibodies recorded a noteworthy abundance, which feature notable logFCs or significance, while not reaching the significance and logFC thresholds simultaneously. The results of the statistical analysis are summarized in the volcano plot (**Figure 1**) and listed in **Supplementary Tables S3** and **S4**.

Post-ECP, the proinflammatory cytokines IL-15, and IL-17, as well as the receptors IL1R2 and TLR3 were increased. Strikingly, the anti-inflammatory cytokine IL-13 was moderately increased post-ECP, while its receptors IL13R1 and IL13R2 were decreased after the ECP. The chemokines CCL7 and CXCL12 were increased, while CXCR5 was decreased upon ECP. Additionally, cell death-relevant proteins like Fas and TRAIL were increased, while FasL was decreased post-ECP.

Moreover, several markers for peripheral blood mononuclear cell populations were altered. Regarding myeloid cells, the soluble forms of CD163, CD14, and CSF1R, were increased post-ECP. ECP increased the levels of CD3deg and altered the expression of proteins involved in T cell adhesion and function like CD44, CD99, CD28, and ICOS. S10A8, involved in regulatory T cell differentiation, was increased post-ECP. Marker for B cells and components of BCR signaling like SLAF8, and CD81 were downregulated post-ECP.

ECP also affected the expression of growth factors and their receptors, as well as cell adhesion and angiogenesis. The growth factor NGF-β was higher post-ECP, while the levels of FGF2, BMP5, NGFR, IGF1R were lower post-ECP. The cell adhesion molecules like Integrins α4, Integrin α6, VCAM1, NCAM1, CEAM1, CEAM5, and the angiogenesis-relevant Angiopoietin 4 were increased upon ECP treatment.

**Figure 2:**
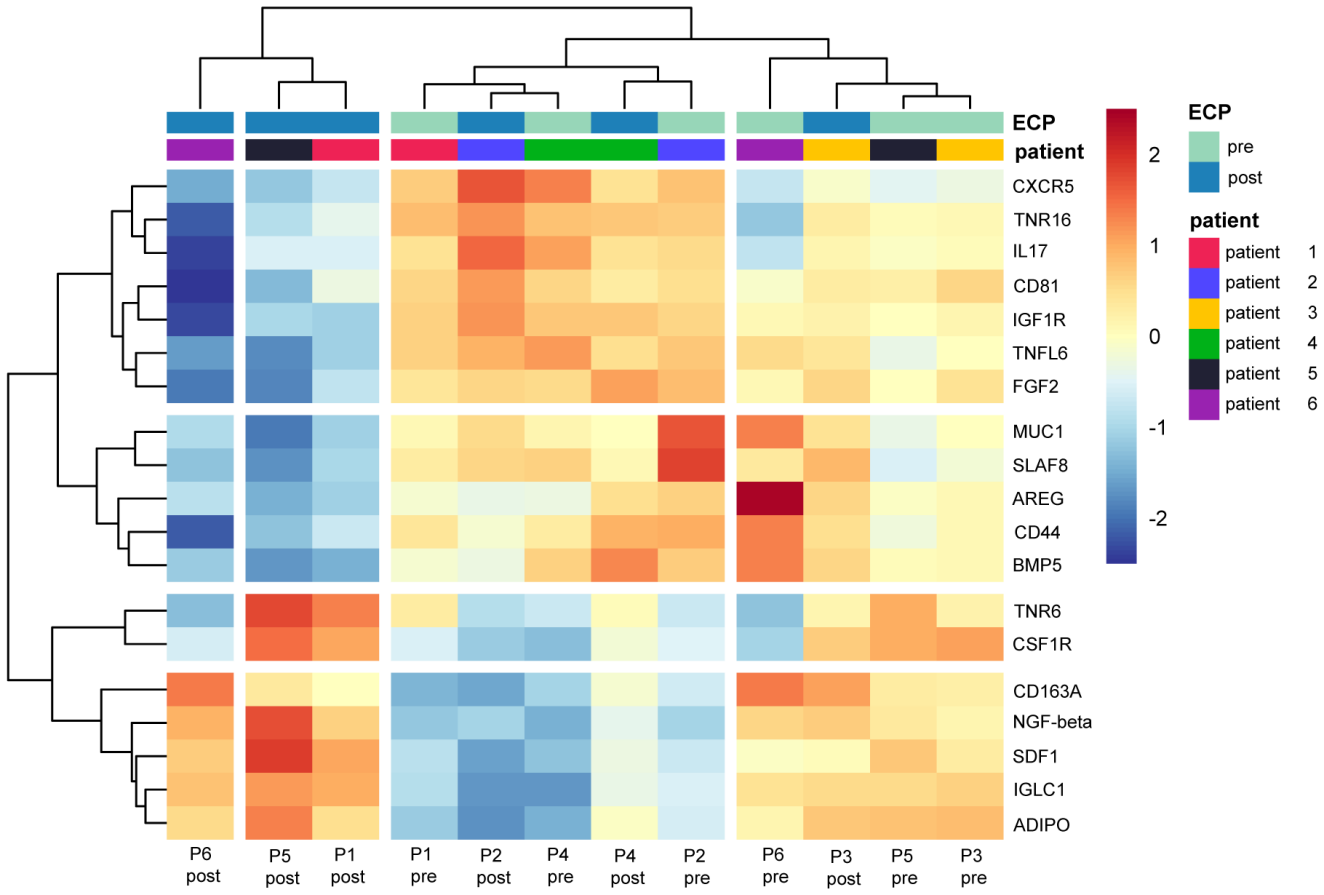
Heatmap displaying the relative expression of proteins identified as differential in the comparison post-ECP vs pre-ECP. Values were centered and scaled by proteins.

The heatmap analysis of the comparisons post-ECP vs. pre-ECP demonstrated distinct heterogeneity between and within the CTCL patients. Relative expression levels for differentially abundant proteins identified in the comparison post-ECP vs pre-ECP are summarized in **Figure 2**.

### 3.2 ECP induces alterations in the composition of the PBMC populations

**Figure 3:**
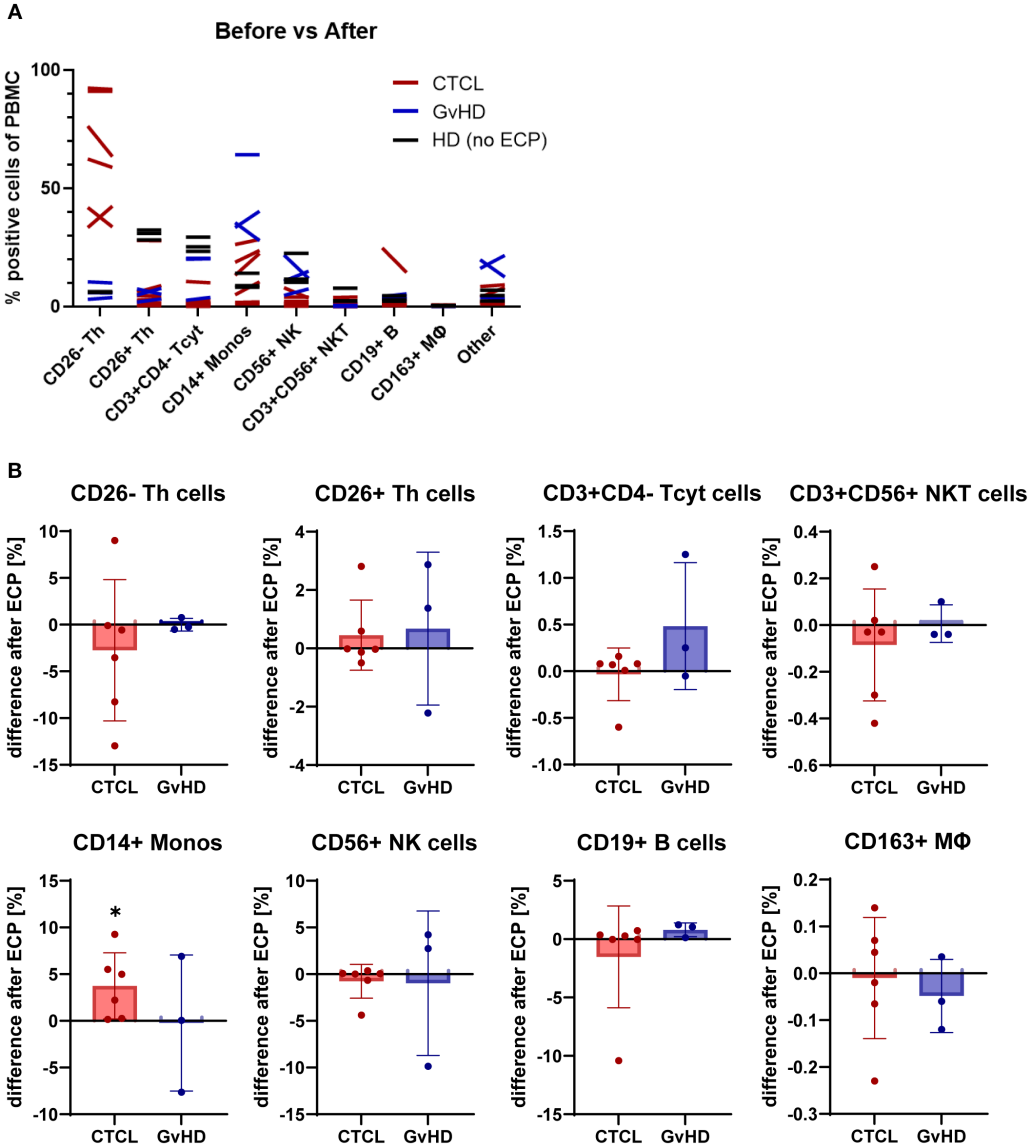
Effect of ECP on PBMC subset frequencies. **(A)** Overview of changes in PBMC subsets from CTCL and GvHD patients before and after ECP in comparison to untreated healthy donors (HD) **(B)** Bar plots show the difference in cell subset frequencies after ECP. Asterisks mark significant changes pre- vs post-ECP.

Initially, the percentage of positive cells of the PBMCs was assessed in CTCL patients, and GvHD patients pre- and post-ECP and compared to healthy controls (**Figure 3A**). Expectedly, the CD4+CD26- Sézary cell population was higher in CTCL patients than in GvHD patients and healthy controls. The CD4+CD26+ T helper cell, and the CD3+CD4- cytotoxic T cell numbers were lower in both CTCL, and GvHD patients compared to healthy controls. Strikingly, the CD14+ monocytes were increased in both CTCL and GvHD patients compared to healthy controls with a tendency for a further increase post-ECP. Regarding CD56+ NK cells, a decreased amount was detected in CTCL patients compared to GvHD patients and healthy donors. The subset of CD3+CD56+ NKT cells, and CD19+ B cells did not differ between CTCL, GvHD patients and healthy donors. In GvHD patients, a higher number of other, in this FACS panel not further specified, PBMC subsets were detected, potentially subsets of dendritic cells.

Subsequently, the difference between pre- and post-ECP regarding the PBMC frequencies were measured (**Figure 3B**). In CTCL, the CD4+CD26- malignant cell population was decreased or stable apart from one outlier. In GvHD, this cell population could be disregarded. The CD3+CD4- cytotoxic T cells tended to be increased post-ECP in GvHD patients but were unaffected in CTCL patients. No alterations in the frequency of the CD4+CD26+ T helper cells, the CD19+ B cells, and the CD3+CD56+ NKT cells were measured in neither CTCL nor GvHD patients.

Concerning the myeloid PBMC populations, a significant increase in CD14+ monocytes and a minor tendency towards an increase in CD163+ monocytes and macrophages were measured in CTCL patients post-ECP. In GvHD patients, mixed effects on the CD14+ monocytes and the CD56+ NK cells were detected. Depending on the individual patient, in- or decreased levels were found post-ECP. In CTCL, the CD56+ NK cell numbers remained stable post-ECP. A detailed analysis of the PBMC subset frequencies pre- and post-ECP is provided in the supplements (**Supplementary Figure S2**).

### 3.3 ECP shapes the expression of pro- and anti-inflammatory cytokines and cell-death mediators in PBMCs

**Figure 4:**
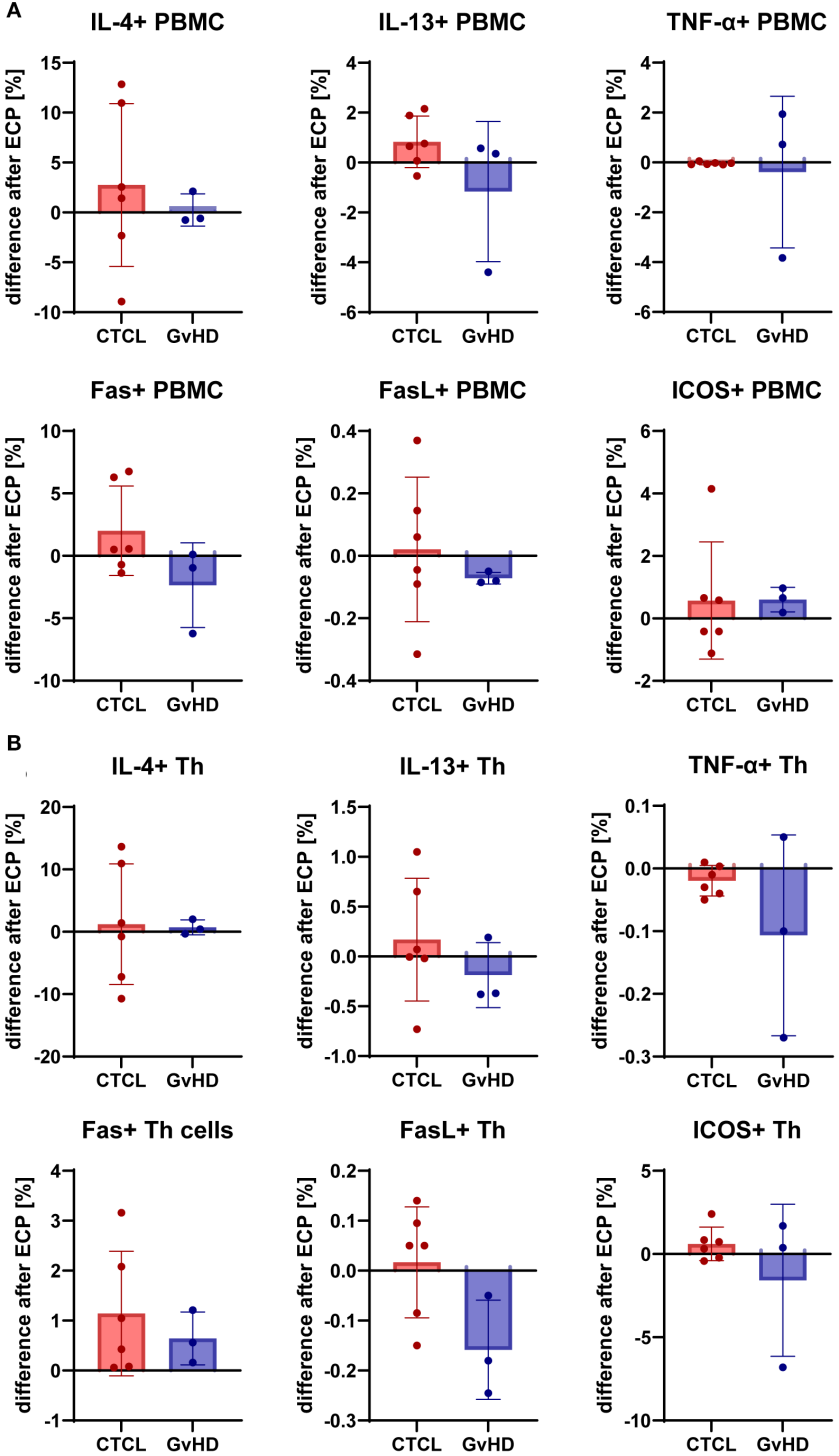
Effect of ECP on expression of pro- (TNF-α) and anti-inflammatory (IL-4, IL-13) cytokines, and immunomodulatory markers like Fas, FasL, and ICOS by PBMC **(A)** and CD4+ T helper cells **(B)** from CTCL and GvHD patients pre and post-ECP.

In addition to the plasma protein screening, the pro-inflammatory molecules TNF-α and ICOS, the Th2 cytokines IL-4 and IL-13, the cell death mediators Fas and FasL were measured in PBMC (**Figure 4A**) and CD4+ T helper cells (**Figure 4B**) pre- and post-ECP with flow cytometry. In the CTCL patients, mixed effects on the expression of IL-4, IL-13, and FasL were measured; both in- and decreased levels were found post-ECP, in contrast to the more consistent patterns detected in plasma. The levels of Fas were increased in PBMCs and even further in Th cells in CTCL.

In GvHD patients, Fas expression in PBMC and FasL expression in PBMC and Th cells were lower, but Fas expression in Th cells was higher post-ECP. IL-13 expression was lower post-ECP, while IL-4 expression remained unchanged. Besides, ICOS expression was decreased in GvHD patients post-ECP in Th cells. In CTCL patients, ICOS levels remained stable, but were increased in the PBMC of one patient post-ECP. Likewise, post-ECP TNF-α levels were unaffected in CTCL patients, and lower in GvHD patients with a more pronounced effect in Th cells than in PBMC.

## 4 Discussion

CTCL patients suffer from numerous immune alterations, including increased secretion of immunosuppressive Th2 cytokines like IL-4 and IL-13 and a decreased proinflammatory Th1-mediated immune response (23–25). Notably, sepsis due to increased susceptibility to infections is a leading cause of lethality in CTCL patients (26). ECP is an immunomodulatory therapy that aims to restore the immune balance in CTCL, as well as in other inflammatory conditions like GvHD or solid organ transplant rejection. However, the exact immunological mechanisms behind the ECP are still only incompletely understood.

In our study cohort, we detected alterations in the composition of the PBMC populations, pro- and anti-inflammatory cytokines and chemokines, cell death inducers, growth factors, and components of the extracellular matrix caused by ECP treatment. Strikingly, those alterations were short-term induced and could be recorded within one cycle of ECP treatment. Another point is that the CTCL patients received different immunomodulatory cotreatments (e.g. Interferon, methotrexate, dimethyl fumarate, and Mogamulizumab) together with ECP. Therefore, the measured effects seem to be independent from the cotreatment and thus, can be attributed to the ECP.

Early publications from the 1990s and 2000s report on the effects of ECP on the production of TNF-α, and IFN-γ, restoration of the Th1/Th2 balance, lymphoid cell death, and lymphocyte immunogenicity (18, 19, 27–29). In the present study, our results regarding the restoration of the Th1/Th2 balance were mixed and characterized by a high patient heterogeneity. These findings could indicate that this phenomenon can be primarily attributed to the long-term effects of the ECP.

The effects of the ECP on the composition of the cell populations in the peripheral blood of CTCL patients has been extensively studied. Ventura et al. discovered that ECP induces a distinct transcriptomic signature in monocytes indicative of dendritic cell maturation (30). In CTCL patients, reduced numbers of NK cells were measured, and long-term ECP treatment led to the recovery of the reduced NK cell subsets (24). Shiue et al. observed an increase in CD8+ cytotoxic T cells upon ECP in CTCL patients who responded well to ECP (10). In our CTCL cohort, we measured a significant increase in the monocyte count, while the NK cells remained stable post-ECP. In the GvHD patients, slightly higher numbers of cytotoxic T cells post-ECP were detected, while in the CTCL patients the cytotoxic T cells remained unaffected.

During the ECP, 10-15% of the peripheral blood lymphocytes are treated and become apoptotic. In brief, during “transimmunization”, the apoptotic cells are processed by antigen-presenting cells, and a tumoricidal immune response is initiated (29, 31). Recently, Lackner et al. found that ECP can induce immunogenic cell death (ICD) in the malignant CTCL cells via activating dendritic cells and thereby enhancing tumor immunogenicity (32). In line with this finding, in our study, the apoptosis-inducing factors Fas and TRAIL were increased post-ECP (33). Our study offers new insights into the therapeutic mechanisms of ECP both in CTCL and in GvHD. Limitations are the small patient cohort with considerable patient heterogeneity, and heterogeneous pharmacological cotreatments. In this study, the short-term effects of only one ECP treatment were investigated, while to date, the long-term effects of ECP after several months of therapy have been more intensively studied (32, 34). The therapeutic response to ECP therapy can be firstly evaluated after six months of therapy (35). This study demonstrates that just one ECP treatment already induces significant immunologic changes in both the plasma cytokine profile and the composition of the PBMC populations, which illustrates the profound impact of the ECP on the immune system. Thereby, we contribute to a more profound understanding of the molecular mechanisms of the ECP. Since an early inclusion of the ECP in the therapeutic regimen of CTCL patients is beneficial, we aim to further improve the therapeutic outcomes of the ECP both as mono- and combination therapy.

## Data Availability

The raw data supporting the conclusions of this article will be made available by the authors, without undue reservation.
