# Supplementary material for: Extracorporeal photopheresis therapy rapidly changes the cytokine profile and tumor microenvironment in cutaneous T cell lymphoma
Source: Front Immunol. 2025 Sep 25;16:1669015. doi: 10.3389/fimmu.2025.1669015 (PMC12507584; doi:10.3389/fimmu.2025.1669015)
Supplement: Supplementary file 1 [file DataSheet1.pdf]

Supplementary Material

1 Supplementary Figures and Tables

1.1 Supplementary Figures

Supplementary Figure 1

Representative gating strategy for peripheral blood mononuclear cells (PBMCs)

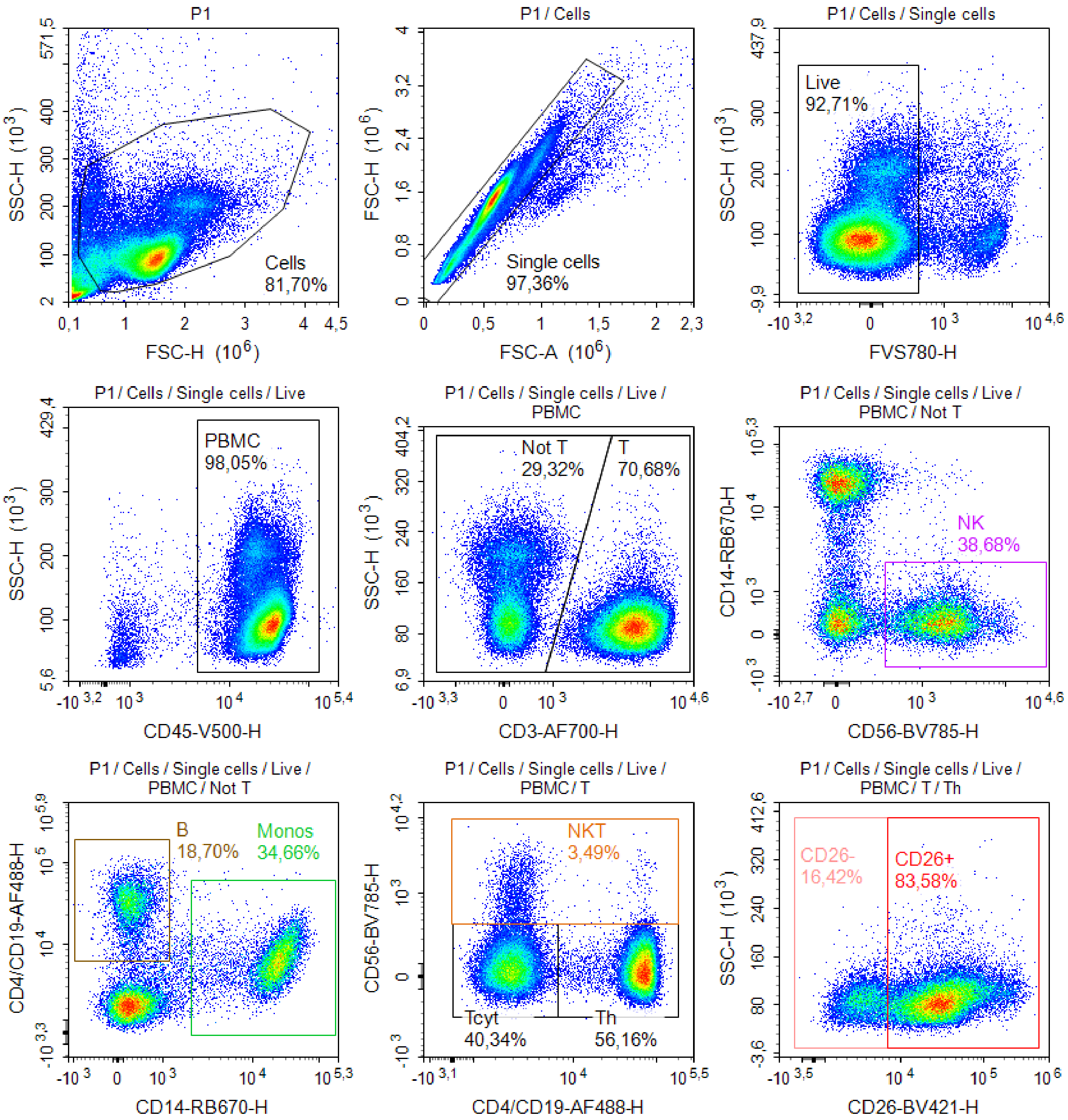

Supplementary Figure 2

PBMC subset frequencies pre- and post ECP treatment

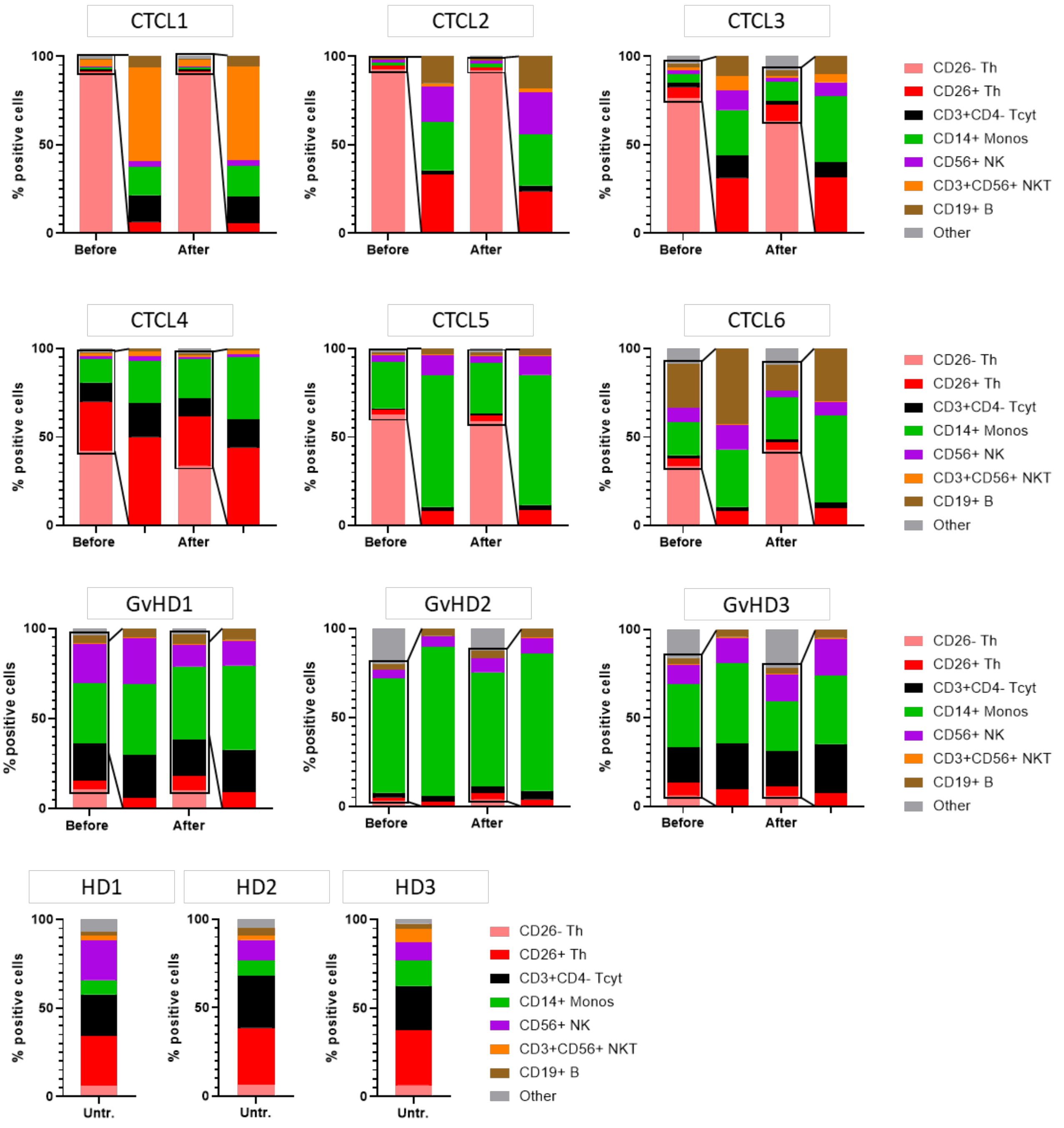

Supplementary Table 1

CTCL patient data. Bexa = Bexarotene; Brent = Brentuximab; DMF = Dimethylfumarate; ECP = Extracorporeal Photophoresis; IFN = Interferone; Moga = Mogamulizumab; MTX = Methotrexate; SC = Sézary cells

Patient	Gender, age	TNM stage	Leuko-cytes/nl	T lympho-cytes [%]	CD4/µl	SC/µl	SC [%]	CD4/CD8	treatment
1	m, 73	T4NxM0B2	18.04	93.8	10070	9810	97.42	44.3	IFN, ECP, DMF, Bexa, PUVA
2	f, 76	T4N3bM0B2	6.30	86.4	1303	1085	83.27	4.8	ECP, DMF, IFN
3	f, 75	T4NxM0B2	10.49	93.1	3139	993	31.63	25.7	ECP, Moga, DMF, IFN, PUVA
4	m, 67	T4N1M0B2	9.74	95.2	2803	2525	90.08	495.5	ECP, DMF, Bexa, MTX, PUVA
5	w, 80	T4N0M0B2	12.6	95.7	5099	4190	82.17	17.8	ECP, Brent, Moga, DMF, TSEB, IFN, Bexa
6	m, 55	T4NxM0B2	15.04	89.8	2256	1680	50.10	3.6	ECP., MTX, Moga
Norm			4.2 - 10.2	60 - 83 %	528 - 1495	< 1000		1 - 2.8	

Supplementary Table 2

Panel 1			
**Antigen**	**Clone**	**Fluorochrome**	**Company**
CD19	SJ25C1	AF488	BioLegend
CD4	OKT4	AF488	BioLegend
IL-4	8D4-8	PE	eBioscience
CD14	63D3.rMAb	RB670	BD Biosciences
IL-13	JES10-5A2	PE-Cy7	BioLegend
TNF-α	MAb11	APC	BioLegend
CD3	SK7	AF700	BioLegend
Live/dead	-	FVS780	BD Biosciences
CD26	M-A261	BV421	BD Biosciences
CD45	2D1	V500	BD Biosciences
CD56	HCD56	BV785	BioLegend

Panel 2			
**Antigen**	**Clone**	**Fluorochrome**	**Company**
CD19	SJ25C1	AF488	BioLegend
CD4	OKT4	AF488	BioLegend
Fas	DX2	PE	BioLegend
CD14	63D3.rMAb	RB670	BD Biosciences
ICOS	DX29	PE-Cy7	BD Biosciences
CD163	RM3/1	APC	BioLegend
CD3	SK7	AF700	BioLegend
Live/dead	-	FVS780	BD Biosciences
CD26	M-A261	BV421	BD Biosciences
CD45	2D1	V500	BD Biosciences

FasL	NOK-1	BV650	BD Biosciences
CD56	HCD56	BV785	BioLegend

Supplementary Table 3

Proteins with differential abundance in post-ECP samples and pre-ECP samples. Proteins with a positive logFC value had a higher abundance in post-ECP samples, proteins with a negative value in pre-ECP samples. In addition, p values adjusted for multiple testing are listed. The Uniprot-Identifier links to the Uniprot-Entry.

**Protein**	**UniprotID**	**logFC**	**AveExp**	**p-value**	**adj.p-val**
IGLC1	P0CG04	1.00	14,79	0,00209	0,0355
ADIPO	Q15848	1.00	14,47	0,00241	0,0385
TNR6	P25445	0,69	12,43	0,00191	0,0335
C163A	Q86VB7	0,66	13,48	0,0028	0,042
NGF-beta	P01138	0,6	11,02	8,98E-09	0,00000458
CSF1R	P07333	0,58	13,74	0,00137	0,0259
SDF1	P48061	0,53	11,69	0,000284	0,0136
BMP5	P22003	-0,52	12,87	0,000363	0,0154
SLAF8	Q9P0V8	-0,53	14,16	0,00267	0,0413
CD81	P60033	-0,54	13,94	0,0000945	0,00688
TNR16	P08138	-0,55	12,67	0,00122	0,0239
FGF2	P09038	-0,56	13,74	0,000968	0,0225
IL17	Q16552	-0,58	10,54	0,000998	0,0225
CD44	P16070	-0.60	13,11	0,000593	0,0172
AREG	P15514	-0,63	13.20	0,00101	0,0225
IGF1R	P08069	-0,67	12,68	0,00114	0,0233
TNFL6	P48023	-0.70	11.50	0,000542	0,0172
CXCR5	P32302	-0,71	13,61	0,00114	0,0233
MUC1	P15941	-0,83	13,24	0,000000287	0,0000733

Supplementary Table 4

Proteins with noteworthy abundance in post-ECP samples and pre-ECP samples, which feature notable logFCs or significance, while not reaching the significance and logFC thresholds simultaneously. Proteins with a positive logFC value had a higher abundance in post-ECP samples, proteins with a negative value in pre-ECP samples. In addition, p values adjusted for multiple testing are listed. The Uniprot-Identifier links to the Uniprot-Entry.

**Protein**	**UniprotID**	**logFC**	**AveExp**	**p-value**	**adj.p-val**
IGKC	P01834	1,04	15,06	0,0051	0,0605
ALBU	P02768	1,03	15,16	0,00426	0,0544
S10A8/9		0,7	12,61	0,00392	0,0513
CO3	P01024	0,7	12,54	0,00476	0,0578
GRN	P28799	0,55	11,09	0,068	0,365
TNF10	P50591	0,51	9,74	0,0175	0,157
ADA17	P78536	0,5	12,21	0,0292	0,232
TFR1	P02786	0,49	11,26	0,000576	0,0172
CD28	P10747	0,45	9,35	0,00000887	0,00113
CD14	P08571	0,44	10,77	0,000761	0,0194
ANGP4	Q9Y264	0,43	11,08	0,00000467	0,000795
IL1R2	P27930	0,38	8,73	0,000394	0,0154
TFR1	P02786	0,36	11,01	0,000567	0,0172
Lactoferin	P02788	0,36	9,63	0,000238	0,0135
ITA4	P13612	0,34	10,24	0,000294	0,0136
CCL7	P80098	0,29	10,66	0,000607	0,0172
PERM	P05164	0,24	9,83	0,0000258	0,00263
ITA6	P23229	0,24	10,21	0,0000559	0,00475
HLA-DR		0,21	9,04	0,00333	0,0472
IL13	P35225	0,18	10,28	0,00239	0,0385
NCAM1	P13591	0,14	9,74	0,00369	0,0495
CNTF	P26441	-0,28	9,16	0,000177	0,0113
TLR3	O15455	-0,37	14,23	0,00142	0,0259
I13R1	P78552	-0,44	11.60	0,00351	0,0484

TGM2	P21980	-0,45	10,08	0,00332	0,0472
I13R2	Q14627	-0,48	13,59	0,000646	0,0173
CD3deg	P07766	-0.50	10,72	0,03	0,235
IL15	P40933	-0,53	9,25	0,0265	0,215
ICOS	Q9Y6W8	-1,05	9,61	0,00665	0,0733
